# Preliminary comparison of the performance of the National Library of Medicine's systematic review publication type and the sensitive clinical queries filter for systematic reviews in PubMed

**DOI:** 10.5195/jmla.2022.1286

**Published:** 2022-01-01

**Authors:** Tamara Navarro-Ruan, R. Brian Haynes

**Affiliations:** 1 navarro@mcmaster.ca, Research Coordinator, Health Information Research Unit, Department of Health Research Methods, Evidence and Impact, McMaster University, Hamilton, Ontario, Canada; 2 bhaynes@mcmaster.ca, Professor Emeritus, Health Information Research Unit, Department of Health Research Methods, Evidence and Impact, McMaster University, Hamilton, Ontario, Canada

**Keywords:** information retrieval, evidence-based medicine, systematic reviews

## Abstract

**Objective:**

The National Library of Medicine (NLM) inaugurated a “publication type” concept to facilitate searches for systematic reviews (SRs). On the other hand, clinical queries (CQs) are validated search strategies designed to retrieve scientifically sound, clinically relevant original and review articles from biomedical literature databases. We compared the retrieval performance of the SR publication type (SR[pt]) against the most sensitive CQ for systematic review articles (CQrs) in PubMed.

**Methods:**

We ran date-limited searches of SR[pt] and CQrs to compare the relative yield of articles and SRs, focusing on the differences in retrieval of SRs by SR[pt] but not CQrs (SR[pt] NOT CQrs) and CQrs NOT SR[pt]. Random samples of articles retrieved in each of these comparisons were examined for SRs until a consistent pattern became evident.

**Results:**

For SR[pt] NOT CQrs, the yield was relatively low in quantity but rich in quality, with 79% of the articles being SRs. For CQrs NOT SR[pt], the yield was high in quantity but low in quality, with only 8% being SRs. For CQrs AND SR[pt], the quality was highest, with 92% being SRs.

**Conclusions:**

We found that SR[pt] had high precision and specificity for SRs but low recall (sensitivity), whereas CQrs had much higher recall. SR[pt] OR CQrs added valid SRs to the CQrs yield at low cost (i.e., added few non-SRs). For searches that are intended to be exhaustive for SRs, SR[pt] can be added to existing sensitive search filters.

## INTRODUCTION

Systematic reviews (SRs) of the medical literature occupy the top echelon of the hierarchy of evidence for consideration by health care decision-makers [1] because they represent an exhaustive summary of the evidence to date concerning health care interventions, diagnostic tests, epidemiology, prognosis, clinical prediction, and economics. If SRs are done well and are up to date (key matters to be determined by the user following retrieval), they summarize the best that science has to offer to inform health care decisions and define the foundation for future research. However, SRs are but a tiny fraction of the health care literature and a subset of all reviews, many of which remain in the traditional narrative mode with arbitrary selection of references and the potential for crippling bias. Thus, accurate and complete retrieval of SRs from large bibliographic databases is important and challenging.

In January 2019, the National Library of Medicine (NLM) inaugurated indexing articles with a new “publication type” [pt] designed to facilitate searches for SRs, distinguishing them from original studies and traditional narrative or clinical reviews, and defined as:

Systematic Review [Publication Type]

A review of primary literature in health and health policy that attempts to identify, appraise, and synthesize all the empirical evidence that meets specified eligibility criteria to answer a given research question. Its conduct uses explicit methods aimed at minimizing bias in order to produce more reliable findings regarding the effects of interventions for prevention, treatment, and rehabilitation that can be used to inform decision making. Year introduced: 2019 [2].

Medical Subject Headings (MeSH), which defines all indexing terms used by the NLM, retains the preceding, more general term, Review[pt]:

Review [Publication Type]

An article or book published after examination of published material on a subject. It may be comprehensive to various degrees and the time range of material scrutinized may be broad or narrow, but the reviews most often desired are reviews of the current literature. The textual material examined may be equally broad and can encompass, in medicine specifically, clinical material as well as experimental research or case reports. State-of-the-art reviews tend to address more current matters. A review of the literature must be differentiated from HISTORICAL ARTICLE on the same subject, but a review of historical literature is also within the scope of this publication type. Year introduced: 2008(1966) [3].

As per MEDLINE policy, indexers are to select terms that describe an article as specifically as possible, so SR[pt] would be applied if an article met the definition for SR[pt]. Furthermore, by indexing policy, an article about the nature, methods, and process of creating a SR (“Systematic review as topic”) would not be indexed using SR[pt] unless it also included a systematic review.

SR[pt] has the potential, if applied accurately, consistently, and in a timely manner, to make the search for SRs easier, with high sensitivity (ability to retrieve SRs) and specificity (ability to filter out articles that are not SRs). However, there are at least three caveats. First, it cannot be assumed that indexers are accurate or consistent [4]. Second, indexing takes time to complete for many journals, averaging 162 days from the publication date [5], so it is less useful for retrieving recently published SRs, which are likely to be the most important, especially for quickly developing topics such as COVID-19. Third, SR[pt] was implemented at the beginning of 2019, and the average useful lifespan of most SRs is longer than that [6], meaning that valid SRs published before 2019 will not be retrieved using SR[pt] on its own.

Clinical queries (CQs) are empirically validated search strategies (also known as hedges or filters) using both text words and MeSH that are designed to retrieve scientifically sound, clinically relevant original and review articles from biomedical literature databases such as MEDLINE with high sensitivity and specificity compared with meticulous, independent hand-searching of full-text journal articles. CQs are tailored for distinct categories of studies (e.g., treatment, prevention, diagnosis, and prognosis), with separate filters for review articles [7], available as an OVID Medline Limit (Clinical Queries>Reviews (maximizes sensitivity)) and on the McMaster Health Knowledge Refinery (McMaster HKR) Projects site [8]. Because CQs focus on key research methods (which do not change appreciably with time), they do not require updating over time [9].

In this investigation, we compared the retrieval performance of SR[pt] with a previously published CQ sensitive for systematic review articles (CQrs), which was validated to have high sensitivity (99.9%) with specificity (52%) [7]. Our purpose was to compare the relative sensitivity (recall) and relative precision of the two filters during a time period when we expected SR[pt] indexing to be in mature operation, twelve to eighteen months after inauguration by NLM and with at least six months' grace for indexing lag following article posting in PubMed.

## METHODS

The key comparisons were verified SR articles retrieved by SR[pt] but not by CQrs (SR[pt] NOT CQrs) and by CQrs but not SR[pt] (CQrs NOT SR[pt]) (Table 1). Articles indexed by both filters were also examined. Searches were conducted in PubMed on March 29, 2021, for PubMed publication dates from January 1, 2020, through June 30, 2020. This time period was chosen to accommodate the lag time between articles being posted to PubMed (pdat) and indexing terms being added. Using StatsDirect Statistical Software (version 3.3.5), we generated random samples of the articles retrieved in each of these comparisons. These were examined by one author to determine if they were valid SRs, applying the SR[pt] definition in MeSH (as noted above) until a consistent pattern became evident. This required direct examination of samples of 50 to 100 articles for each comparison to provide 95% confidence intervals (CIs) of about 10% or less on estimates of performance. Titles of articles were not treated as adequate sources and were ignored in this assessment. Abstracts were perused and, if there was any doubt, full-text articles, if accessible and in English, were read until a decision could be made about whether the article reported a SR. Non-English articles without abstracts were excluded. To verify the reproducibility of this single assessment, we conducted two trial runs with independent assessment by both authors of thirty randomly selected articles, showing 100% agreement in determining whether a retrieved article met the SR[pt] definition.

**Table 1:** Search strategies

Search Filter	Strategy
SR[pt]	Systematic review[publication type]
Clinical Query sensitive filter for review articles (CQrs)	search*[Title/Abstract] OR meta-analysis[Publication Type] OR meta analysis[Title/Abstract] OR meta analysis[MeSH Terms] OR review[Publication Type] OR diagnosis[MeSH Subheading] OR associated[Title/Abstract]

## RESULTS

The SR[pt] filter retrieved only 3.53% of the articles retrieved by CQrs (Table 2). Nevertheless, the SR[pt] NOT CQrs comparison shows that SR[pt] retrieved a small but rich load of SRs not retrieved by CQrs (number needed to read (NNR) to find a valid SR=1.27).

**Table 2:** Search results for various search combinations

Search for 2020/01/01[pdat]: 2020/06/01 [pdat]	Total no. of articles retrieved	Sample of articles validated (n)	No. (% and 95% CI) of articles meeting SR[pt] definition	NNR to find one additional SR
SR[pt]	9,307			
CQrs	263,334			
SR[pt] NOT CQrs	1,028	100	79 (79%, CI 70-87)	1.27
CQrs NOT SR[pt]	253,613	50	4 (8%, CI 2-19)	12.5
CQrs AND SR[pt]	8,309	50	46 (92%, CI 81-98)	1.09

The many articles retrieved by CQrs NOT SR[pt] were diluted for valid SRs (8% SRs, NNR=12.5). Despite this, the large number of articles in the CQrs NOT SR[pt] net (n=253,613) means that over 20,000 SRs would be missed, more in total than captured by the SR[pt] AND CQrs conjunction (8,309 articles). However, SR[pt] AND CQrs contained 89.3% of the articles retrieved by SR[pt], and the intersection of SR[pt] AND CQrs had within it the highest proportion of valid SRs (92%, NNR=1.09).

Furthermore, one can estimate from Table 2 that about 91% of the 9,307 articles retrieved by SR[pt] would meet the SR[pt] definition (i.e., 79% of SR[pt] NOT CQrs + 92% of CQrs AND SR[pt], divided by SR[pt]).

## DISCUSSION

Our comparison shows that over 90% of SR[pt] retrievals of articles meet the MeSH definition of SRs and that few articles retrieved by SR[pt] are not SRs (high specificity and precision). However, SR[pt] is not yet being applied to all SRs. This could be for several reasons. First, delays in indexing for many journals are longer than the six months we allowed [4]. Second, depth of indexing may vary for journals, a trade-off between completeness of the MEDLINE collection and the resources for complete indexing, that is not shared by search filters that include text word terms for screening article titles and abstracts (e.g., CQrs). On the other hand, CQrs missed some valid SRs retrieved by SR[pt], indicating that a comprehensive search would be supported by using both approaches (i.e., CQrs OR SR[pt]). Thus, SR[pt] should be used in conjunction (“OR”ed) with a validated Boolean search filter for systematic reviews, and the articles retrieved by SR[pt] but not CQrs should also be examined.

This project has some limitations. First, the assessment of whether an article was a SR was unblinded as to retrieval search (SR[pt] or CQrs) and completed by only one reviewer. Second, we tested the performance of both approaches for only one relatively recent time period. While more contemporary comparisons (e.g., most recent six months) would result in lower performance for SR[pt] due to indexing delays, delayed comparisons (e.g. beyond six months) would also result in lower performance for SR[pt] unless back-indexing is done for articles published before SR[pt] was introduced. Third, we examined only limited random samples of articles retrieved, leaving the estimates somewhat imprecise. For example, the estimate of SRs of 79% of 100 articles retrieved by SR[pt] but not CQrs has a 95% CI of 69.7% to 86.5%, which we offer as adequate to document the conclusion that SR[pt] provides value-added benefits in this respect to CQrs, a sensitive, validated search filter.

For users seeking the most recent SRs, using SR[pt] alone would not be adequate for finding recently published SRs (because they will not have been indexed yet) or for finding SRs published before 2019. Both conditions can problematic. For quickly evolving topics such as COVID-19, missing current SRs can impede both research and application of pertinent evidence in health care. For topics for which the most recent reviews were published before 2019, searching with SR[pt] alone would fail. This could be overcome by retrospectively indexing, going back at least to match the usual lifespan of a review, previously estimated at a median of 5.5 years [6]. Discussions for whether it would be “worth it” to do so need to take into account the current waste in ill-informed research and the cost and harm of using outdated evidence in health care [10].

Our research is preliminary, and many additional studies could be undertaken given the promise SR[pt] shows in this investigation. For example, its sensitivity and specificity could be measured directly in conjunction with hand-searching of journals or relative to other search standards such as that of the Cochrane Collaboration.

In conclusion, the addition of indexing for SRs in PubMed is welcome and adds SRs not retrieved by a validated, sensitive Boolean search filter. However, at present, it can only play a limited, adjunctive role for comprehensive searches for SR.

## Data Availability

Data associated with this article are available in the Open Science Framework at <https://osf.io/fdwk8/?view_only=b740b8c0592c4e079452a6bf2865eec2>.
